# Activation of Melanocortin Receptors MC_**1**_ and MC_**5**_ Attenuates Retinal Damage in Experimental Diabetic Retinopathy

**DOI:** 10.1155/2016/7368389

**Published:** 2016-01-12

**Authors:** S. Rossi, R. Maisto, C. Gesualdo, M. C. Trotta, F. Ferraraccio, M. K. Kaneva, S. J. Getting, E. Surace, F. Testa, F. Simonelli, P. Grieco, F. Merlino, M. Perretti, M. D'Amico, C. Di Filippo

**Affiliations:** ^1^Multidisciplinary Department of Medical-Surgical and Dental Specialties, Second University of Naples, 80138 Naples, Italy; ^2^Department of Experimental Medicine, Second University of Naples, 80138 Naples, Italy; ^3^Department of Clinical, Public and Preventive Medicine, Second University of Naples, 80138 Naples, Italy; ^4^The William Harvey Research Institute, Barts and The London School of Medicine, Queen Mary University of London, London EC1M 6BQ, UK; ^5^Faculty of Science and Technology, Department of Life Sciences, University of Westminster, London W1W 6UW, UK; ^6^Department of Translational Medicine, University of Naples Federico II, 80131 Naples, Italy; ^7^Pharmacy Department, University of Naples Federico II, 80131 Naples, Italy

## Abstract

We hypothesize that melanocortin receptors (MC) could activate tissue protective circuit in a model of streptozotocin- (STZ-) induced diabetic retinopathy (DR) in mice. At 12–16 weeks after diabetes induction, fluorescein angiography (FAG) revealed an approximate incidence of 80% microvascular changes, typical of DR, in the animals, without signs of vascular leakage. Occludin progressively decreased in the retina of mice developing retinopathy. qPCR of murine retina revealed expression of two MC receptors,* Mc1r* and* Mc5r*. The intravitreal injection (5 *μ*L) of the selective MC_1_ small molecule agonist BMS-470539 (33 *μ*mol) and the MC_5_ peptidomimetic agonist PG-901 (7.32 nM) elicited significant protection with regular course and caliber of retinal vessels, as quantified at weeks 12 and 16 after diabetes induction. Mouse retina homogenate settings indicated an augmented release of IL-1*α*, IL-1*β*, IL-6, MIP-1*α*, MIP-2*α*, MIP-3*α*, and VEGF from diabetic compared to nondiabetic mice. Application of PG20N or AGRP and MC_5_ and MC_1_ antagonist, respectively, augmented the release of cytokines, while the agonists BMS-470539 and PG-901 almost restored normal pattern of these mediators back to nondiabetic values. Similar changes were quantified with respect to Ki-67 staining. Finally, application of MC_3_-MC_4_ agonist/antagonists resulted to be inactive with respect to all parameters under assessment.

## 1. Introduction

Diabetic retinopathy is a leading cause of adult blindness and is the most common complication of diabetes. It affects more than 90% of people with diabetes, ultimately leading to retinal edema, neovascularization, and, in some patients, vision loss [1, 2]. Systemic control of blood glucose can slow down the progression of diabetic retinopathy but fails to stop or reverse clinical signs of it [3, 4]. Hence understanding the molecular pathways governing the pathophysiology of DR and targeting them is essential to the prevention of catastrophic visual loss arising from vision-threatening complications of diabetic retinopathy such as macular edema, vitreous hemorrhage, and tractional retinal detachment.

Melanocortins are endogenous peptides that possess a wide range of biological activities, including inhibition of leukocyte activation, promotion of inflammation resolution, and the ensuing tissue protection [5–12]. These effects on the immune response are brought about by five distinct melanocortin receptors, termed from MC_1_ to MC_5_, which are ubiquitously expressed except for the MC_2_ which is localised to the adrenal glands [13]. Within the eye, MC_3_, MC_4_, and MC_5_ are expressed in the inner neural retinal layers [14, 15], with MC_3_ and MC_4_ expression being reported also in the layer of retinal ganglion cells [14, 15]. MC_5_ alone has been detected in the neural outer plexiform layer, whilst MC_1_ and MC_5_ are detected in retinal pigment epithelial cells [16, 17]. There is scant knowledge on the biology associated with these receptors in the eye. Work is limited to the most common melanocortin peptide, *α*-melanocyte stimulating hormone (*α*-MSH), which activates all MC receptors (except MC_2_), controls the development and neurotrophism of the ocular tissues [18–20], and exerts protective effects on the retinal vascular endothelial cells [21, 22].

The present study aimed at establishing the efficacy of melanocortin peptides in the prevention of DR and characterizing the MC subtypes engaged. We made use of a mouse model of STZ-induced DR, an experimental system suitable for replicating the early signs of nonproliferative DR, such as loss of retinal pericytes and capillaries, thickening of the vascular basement membrane and increased vascular permeability [23]. Using a combination of biochemical and functional analyses, we identify novel therapeutic circuits in DR mediated by specific MC receptors.

## 2. Materials and Methods

Streptozotocin was purchased from Sigma-Aldrich (city, country), MTII from Bachem Ltd. (Saffron Walden, Essex, UK), and SHU9119 from Phoenix Pharmaceuticals (Karlsruhe, Germany). Other compounds were supplied (BMS-470539, AGRP) or synthetized (PG-901, PG20N) by Professor Grieco (University of Naples Federico II). All compounds were stored at −20°C before use and dissolved in sterile PBS, pH 7.4.

### 2.1. Animals and DR

This study was performed according to the guidelines of the Ethic Committee for animal experiments at the Second University of Naples. C57BL/6 mice (Harlan, Italy) aged 7 to 10 weeks were rendered diabetic with one intraperitoneal injection of STZ (65 mg/kg, Sigma-Aldrich, St. Louis, MO, USA) freshly dissolved in 10 mM citrate buffer (pH 4.5). Development of diabetes (defined by blood glucose greater than 250 mg/dL) was verified 1 week after the STZ injection (Glucometer Elite XL; Bayer Corp., Elkhart, IN). Blood glucose levels were checked intermittently throughout the study in order to confirm the maintenance of the diabetic condition.

C57BL/6 mice were divided into 8 groups (*n* = 10 animals per group), labelled consecutively from 1 to 10 to repeat the fluorescein angiography (FAG) to the same animal at each time point considered. Mice were randomised into the following experimental groups: (1) nondiabetic mice; (2) diabetic mice; (3) diabetic mice treated with intravitreal injection of the MC_1_ receptor agonist BMS-470539 [24]; (4) diabetic mice treated with intravitreal injection of the mixed MC_3_-MC_4_ receptor agonist MTII [25]; (5) diabetic mice treated with MC_1_ receptor antagonist agouti related protein (AGRP; [26]); (6) diabetic mice treated with intravitreal, MC_5_ agonist PG-901 [26]; (7) diabetic mice treated with intravitreal MC_3_-MC_4_ receptor antagonist SHU9119, [27]; (8) diabetic mice treated with intravitreal MC_5_ receptor antagonist PG20N, [28].

In all cases, animals were monitored over a 16-week period for the development of diabetes, with specific analyses at weeks 8, 12, and 16 when fluorescein angiography was conducted. At the end of each time course the animals were sacrificed and the eye ball was displaced forward by placing curved forceps around the posterior part and cut in two halves. On one half of each eye the cornea was cut using a sharp blade or scalpel, and the retina was squeezed through the cut together with residual pigment epithelium and lens by applying gentle pressure with the forceps. Dissected retina was placed in cooled PBS, freed from nonretinal tissue using the forceps, and immediately frozen in liquid nitrogen and stored at −80°C for subsequent biochemical analysis. The other half of each eye was fixed by immersion in 10% neutral buffered formalin and paraffin-embedded for immunohistochemistry.

*Intravitreal Injections*. Seven days after the development of diabetes the mice were anesthetized by pentobarbital (45 mg/kg in saline). Tropicamide (5%) was instilled into the right eye of each animal, in order to induce dilatation of pupils, and tetracaine (1%) was injected for local anaesthesia. Physiological saline or MC receptor ligand preparations (5 *μ*L volume) were administered via intravitreal injection into the right eye using sterile syringes fitted with a 30-gauge needle (Microfine; Becton Dickinson AG, Meylan, France), as previously described [23]. The following MC receptor ligands were used, at the indicated dose as selected from the reported publications: BMS-470539, 33 *μ*mol [24]; MTII, 9.3 nmol [25]; SHU 9119, 9 nmol [27]; PG-901, 7.32 nM [29]; PG20N, 130 nM [28]; agouti related protein or AGRP, 1 *μ*g [27]. Each compound was injected every 4 weeks from the development of diabetes.

### 2.2. Fluorescein Angiography (FAG)

FAG was performed by using a Topcon TRC-50DX apparatus (Topcon, Tokyo, Japan) following intraperitoneal injection of 10% fluorescein sterile solution (1 mL/kg body weight, AK-Fluor; Akorn, Inc.). Fundus photographs were captured in order to display the retinal vasculature and to evaluate the early typical alterations of diabetic microangiopathy.

### 2.3. EIA Assay for Endogenous *α*-MSH within the Retina

A commercial kit (Phoenix Pharmaceuticals, Inc., Karlsruhe, Germany) was used following the manufacturer protocol in order to assess the levels of the protein within the retina of nondiabetic and diabetic mice with retinopathy.

### 2.4. Real Time PCR

Total RNA was extracted using RNeasy Plus Mini Kit (Qiagen, West Sussex, UK), according to the manufacturer's instructions. Contaminating DNA was removed from RNA preparations using the Ambion Turbo DNA-free system (Life Technologies, Paisley, UK) using manufacturer's instructions. The concentration and purity of the RNA were then analysed using the Nandrop ND-1000 (NanoDrop Technologies, Wilmington, DE). Complementary DNA (cDNA) was obtained by reverse transcription (RT) of 1 *μ*g of total DNase-treated RNA, using the Superscript III Reverse Transcriptase System (Invitrogen, Carlsbad, CA, USA) and oligo(dT) primers following manufacturer's protocol.

Conventional PCR was performed for detecting the expression of murine* Mc1r, Mc3r,* and* Mc5r* genes using cDNA (150 ng/reaction), specific primers (Quantitect Primer Assays, Qiagen, West Sussex, UK), and Thermo Scientific 1.1x ReddyMix PCR Master Mix (Life Technologies, Paisley, UK). The following amplification profile was applied: 95°C for 2 min; 35 cycles—94°C for 30 s, 55°C for 35 s, and 72°C for 65 s, followed by final elongation step at 72°C for 5 min.

Melanocortin receptor expression was quantified using the predesigned Quantitect Primers (ABI Prism 7900 Sequence Detection System; Applied Biosystems Inc.) and 2x Power SYBR Green Mastermix (Applied Biosystems, Thermo Fisher Scientific Inc., Paisley, UK). The absence of unspecific products was confirmed by analyzing the included dissociation end step. Cycle threshold (Ct) values were measured and calculated by the Sequence Detector software. Relative amounts of mRNA in diabetic retinas were normalized to endogenous control (*Gapdh*) and to the healthy controls. Relative mRNA contents were calculated using the *x* = 2^−ΔΔCt^ equation.

### 2.5. Western Blotting Analysis

Western blot was performed on the retinal tissues 16 weeks after the onset of diabetes, monitoring the M2 marker, the mannose receptor CD206, and the M1 marker, the integrin *α*X/CD11c, according to Di Filippo et al. [30]. Retinal samples were homogenized on ice using RIPA buffer (Santa Cruz Biotechnology, Milan, Italy), containing a protease inhibitor cocktail tablet (Roche Diagnostics, Mannheim, Germany). Lysates were centrifuged at 12,000 g and the supernatant was collected. Protein concentrations were determined using the Bio-Rad protein assay (Bio-Rad Laboratories, Milan, Italy). A total of 100 *μ*g of proteins were separated on denaturing 8% SDS-PAGE and transferred to PVDF membrane. The following primary antibodies were used: anti-M2 mannose receptor (CD206) (1 : 400, Abcam, Cambridge, UK), anti-M1 Integrin Alpha X/CD11c (1 : 200, Bioss, USA). Donkey anti-rabbit polyclonal IgG (Abcam, Cambridge, UK) and goat anti-mouse polyclonal IgG (Santa Cruz Biotechnology, USA) secondary antibodies were used at concentration of 1 : 1000 and 1 : 2000, respectively.

### 2.6. Cytokines Array

A specific kit (ARY006, R&D Systems, Abingdon, UK) was used for the simultaneous measurement of the production of a number of pro- and anti-inflammatory cytokines and chemokines from mouse retinas.

### 2.7. Enzyme-Linked Immunosorbent Assay (ELISA)

To assess the levels of occludin and vascular endothelial growth factor (VEGF) in the retina of diabetic mice, the Quantikine ELISA kits (R&D Systems, Abington, UK) were used, according to the manufacturer's protocol.

### 2.8. Immunohistochemistry

Ocular tissue sections (5 *μ*m) were serially cut from paraffin-embedded tissue and labelled for the detection of Ki-67 by immunohistochemistry, according to previous published protocol [31]. Briefly, sections were incubated with primary mouse monoclonal anti-ki67 (PP-67) antibody (dilution 1 : 250, Abcam, Cambridge, UK) for 30 min at room temperature. Sections were then washed with PBS and incubated with biotin-conjugated goat anti-mouse IgG secondary antibodies and avidin-biotin peroxidase complex (DBA, Milan, Italy).

### 2.9. Statistical Analysis

For the* in vivo* experiments, all values are expressed as mean ± SEM of *n* = 10 mice. Statistical analyses were assessed either by Student's *t*-test (when only two groups were compared) or one-way analyses of variance (ANOVA), followed by Dunnett's* post hoc* test (more than two experimental groups). A probability of *p* < 0.05 was considered sufficient to reject the null hypothesis.

## 3. Results

Intraperitoneal injection of STZ (65 mg/kg) to C57BL/6 mice caused an elevation of the glycemia levels, which remained almost constant throughout the duration of the 16-week observation (Table 1). These levels were not affected by drug treatment at any of the time points under investigation. In contrast, the endogenous levels of *α*-MSH within the retina were significantly reduced after 16 weeks of diabetes (nondiabetic, 72 ng/mL ± 7; diabetic, 27 ng/mL ± 11).

### 3.1. STZ-Induced Diabetes Causes Structural and Microvascular Changes in Mouse Retinas

**Figure 1:**
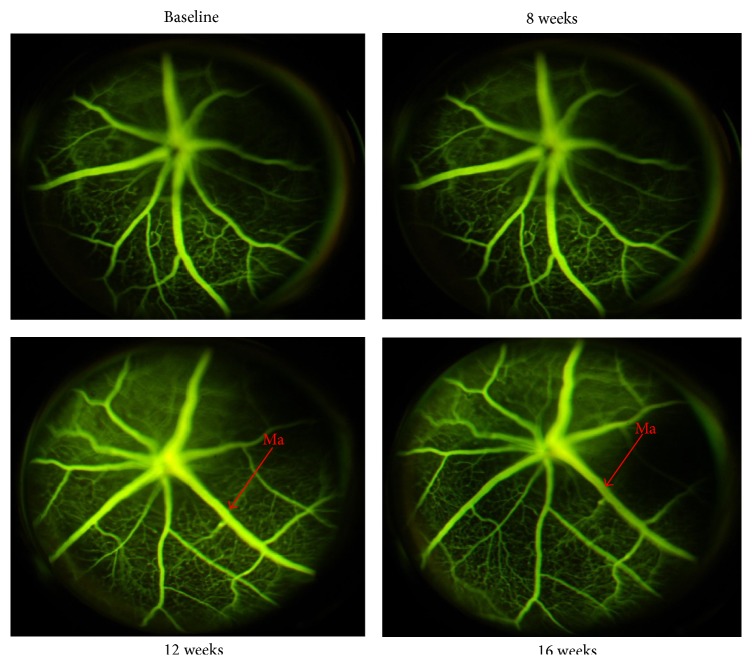
Representative images of FAG performed at baseline and after 8, 12, and 16 weeks from diabetes induction. There were no alterations worthy of note at 8 weeks. At 12 weeks from diabetic induction there was an increase in vascular tortuosity with some microvascular changes such as microaneurysms that were also evident after 16 weeks of diabetes. Each group consisted of 10 mice in which 8 developed clear signs of retinopathy. Ma = microaneurysms.

Fluorescein angiography (FAG) analysis performed over the 16 weeks of diabetes showed structural changes in the retinal vessels with an increased vascular tortuosity and microvascular changes in 8 of the 10 mice analyzed (data at week 12). These alterations became more evident at week 16 after STZ. Indeed, an irregular retinal vessel caliber and microaneurysms were seen at both time points (Figure 1). No signs of deviation from the normal retina vascularization were seen after 8 weeks of diabetes (Figure 1).

### 3.2. Melanocortin Receptors Are Expressed in the Retina of Mice Suffering from Diabetic Retinopathy

**Figure 2:**
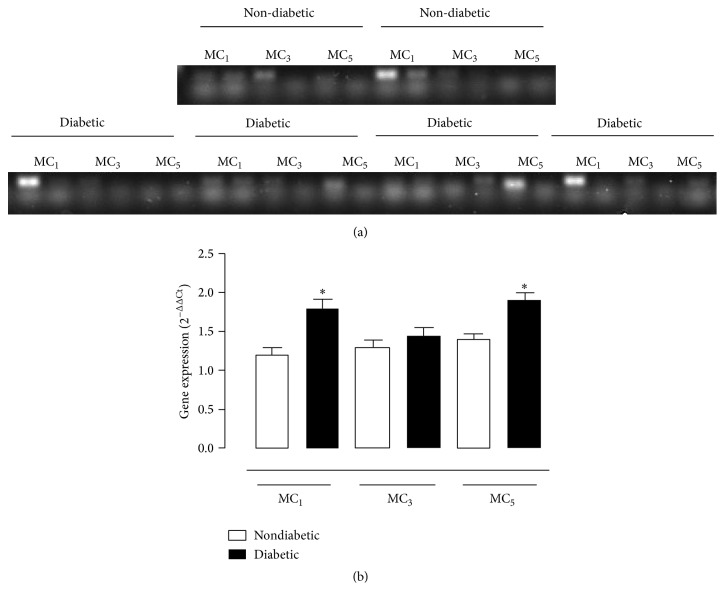
Real Time PCR for the expression of melanocortin receptor subtypes within the retina. (a) Representative traces of the RT-PCR and (b) relative 2^−ΔΔCt^ gene expressions for MC_1_, MC_3_, and MC_5_ receptors assayed after 16-week follow-up in nondiabetic mice, and diabetic mice with retinopathy. Total RNA was extracted using RNeasy Plus Mini Kit and commercially available primer for amplification of mouse MC_1_, MC_3_, and MC_5_ receptors. Negative controls were either RT without enzyme or PCR without cDNA template.

Next we determined the expression patterns of selected MC receptor expression in the retina of diabetic mice that had developed retinopathy, with a focus on MC_1_, MC_3_, and MC_5_ receptors since implicated in the process of inflammation and tissue protection. By conventional PCR we could detect the MC_1_ and MC_5_ signals, but not the MC_3_ (Figure 2). Using qPCR, MC_1_ and MC_5_ displayed a plastic response to diabetes, with elevated expression being quantified by week 16 (Figure 2).

### 3.3. Melanocortin Receptor Activation Modulates the Development of Diabetic Retinopathy

**Figure 3:**
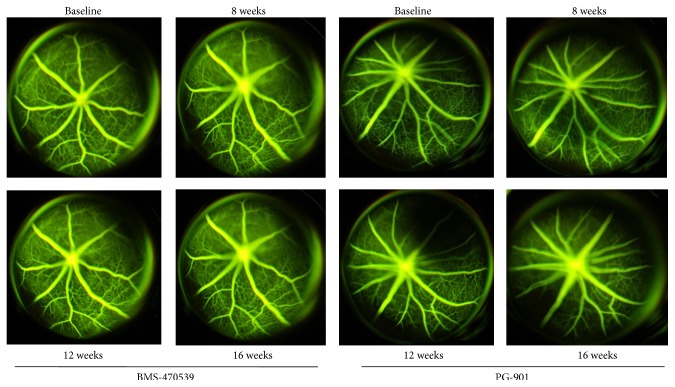
Representative pictures of FAG showed a regular course and caliber of retinal vessels without microvascular changes or vessel leakage at every time point following intravitreal injection of the MC_1_ melanocortin receptor agonist BMS-470539 and of the MC_5_ agonist PG-901. The number of mice for each group was *n* = 10 nondiabetic mice (baseline) and 8 diabetic mice with retinopathy.

Intravitreal injections of the MC_1_ receptor agonist BMS-470539 (33 *μ*mol) or MC_5_ receptor agonist PG-901 (7.32 nM) decreased retinal damage, as demonstrated by FAG. Indeed, regular course and caliber of retinal vessels without microvascular changes or vessel leakage were present at each time point considered after intravitreal injection of MC_1_ receptor agonist BMS-470539 and MC_5_ receptors agonists PG-901, as compared to the untreated diabetic mice with retinopathy (Figure 3).

**Figure 4:**
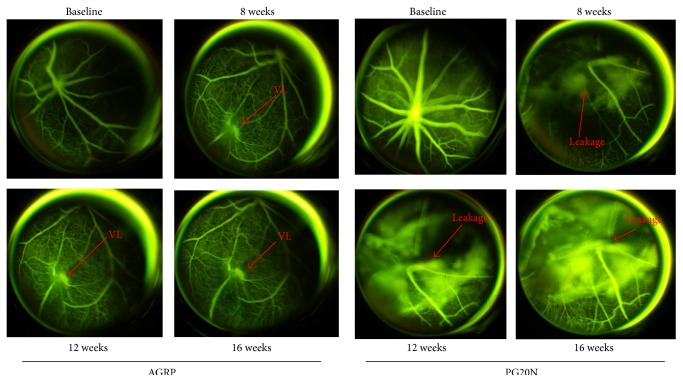
FAG performed at baseline (nondiabetic) and after 8, 12, and 16 weeks from diabetes induction after the intravitreal injection of the MC_1_ receptor antagonist AGRP, and the MC_5_ receptor antagonist PG20N. At baseline no vascular alterations were present 8 weeks following diabetic induction, FAG depicted an increased vascular tortuosity with hyperfluorescent area due to the presence of a venous loop in the retinal inferior nasal area. The first retina damage appears 8 weeks following diabetic induction and was characterized by an extensive hyperfluorescent area of vascular leakage. At 12 and 16 weeks this hyperfluorescent area was extended with progressive dye diffusion. The number of mice for each group was *n* = 10 nondiabetic mice and 8 diabetic mice with retinopathy. VL = venous loop.

**Figure 5:**
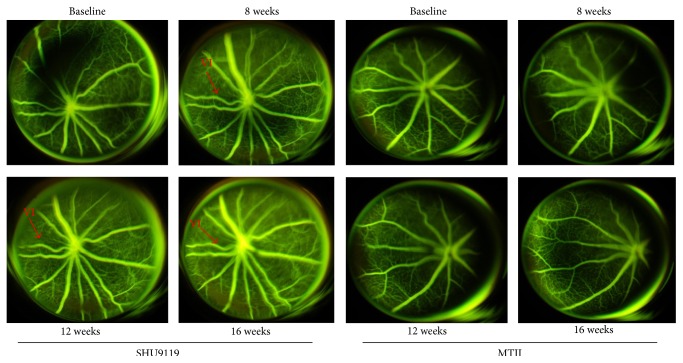
Representative FAGs after intravitreal MC_3_-MC_4_ receptor antagonist SHU9119, and MTII, MC_3_-MC_4_ receptor agonist in diabetic mice with retinopathy. Evident was a progressive increase of the vessel irregularity during the follow-up without microvascular abnormalities or vessel leakage. The number of mice for each group was *n* = 10 nondiabetic mice (baseline) and 8 diabetic mice with retinopathy. VI = vascular irregularity.

To investigated a potential involvement of the MC pathway by endogenous peptides, we tested the effect of receptor antagonists. Intravitreal injection of PG20N (MC_5_ antagonist) and AGRP (MC_1_ antagonist) worsened the retinal injury with evident changes already after 8 weeks after induction of diabetes. Due to the presence of a venous loop (Figure 4) and an extensive retinal vessel leakage with progressive dye diffusion (Figure 4) at 16 weeks after induction, hyperfluorescent areas were observed after treatment with either of the two compounds. Of interest, intravitreal injection of molecules that activate MC_3_, like the agonist MTII (dual MC_3_-MC_4_ agonist) or the antagonist SHU9119 (dual MC_3_-MC_4_ antagonist), did not produce changes of the microvascular bed into the retina of diabetic mice (Figure 5).

### 3.4. Melanocortin Receptor Activation Modulates Levels of Retinal Occludin

**Figure 6:**
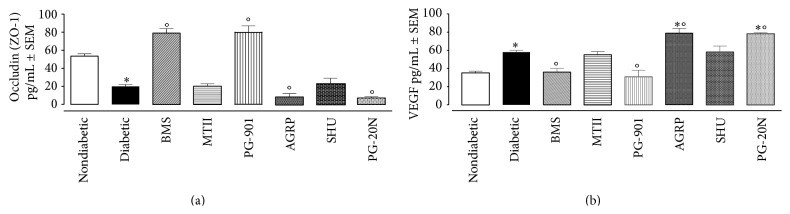
Occludin and vascular endothelial growth factor levels into the retina of STZ-diabetic mice. A Quantikine ELISA kit was used in order to assay after 16 weeks of diabetes the levels of occludin into the retina of nondiabetic and diabetic mice following intravitreal administration of selective melanocortin receptor agonists/antagonists: MC_1_ receptor agonist BMS-470539; MC_3_-MC_4_ melanocortin receptor agonist MTII; MC_1_ receptor antagonist agouti related protein (AGRP); MC_5_ melanocortin receptor agonist PG-901; MC_3_-MC_4_ melanocortin receptor antagonist SHU9119; MC_5_ melanocortin receptor antagonist PG20N. The values represent the mean ± SEM of 8–10 observations. Significant differences against nondiabetic mice are expressed as ^*∗*^ *p* < 0.01. Significant differences versus diabetic are expressed as °*p* < 0.01.

In diabetic mice suffering from retinopathy, cellular tight junctions were damaged as demonstrated by the low levels of occludin detected in retinal homogenates (Figure 6). Of interest, levels of this cell junction marker progressively decreased with the development of retinopathy, reaching the lowest level detected at week 16 after STZ (Figure 6). Such a nadir was further reduced following intravitreal injection of the MC_1_ and MC_5_ receptor antagonist AGRP and PG20N, respectively, over the different time points (Figure 6). Conversely, administration of BMS-470539 or PG-901 increased the retinal occludin levels at 8 weeks, 12 weeks (data not shown), and 16 weeks, compared to untreated diabetic mice (*p* < 0.01, Figure 6), demonstrating a protective effect downstream activation of MC_1_ or MC_5_, respectively. Modulation of MC_3_ signals was without effects (Figure 6).

### 3.5. Melanocortin Receptor Activation Modulates Levels of VEGF in the Retina of Diabetic Mice

As we observed profound alterations in the microcirculation, visual images complemented by the loss of tight junction proteins (of which occludin was selected as faithful marker), we then measured expression of a fundamental angiogenic factor. Retinal levels of VEGF were increased (+66 ± 3%) in diabetic mice suffering from retinopathy at 16 weeks after STZ (*p* < 0.01 versus nondiabetic; Figure 6). These levels were further increased following intravitreal injection of the MC_5_ antagonist PG20N (+35 ± 2.2% on top of diabetic values, *p* < 0.01 versus diabetic; Figure 6) or MC_1_ antagonist AGRP (+36.2 ± 1.8%, *p* < 0.01 versus diabetic; Figure 6). Agonism at MC_1_ or MC_5_ decreased levels of VEGF in the retina of diabetic mice back to levels detected in retinas of untreated diabetic mice (*p* < 0.01; Figure 6).

MC_3_-MC_4_ appear not be involved in this protective effect, as the compounds MTII and SHU9119 failed to modulate VEGF levels assayed during the development of retinopathy in diabetic mice (*p* > 0.05; Figure 6).

**Figure 7:**
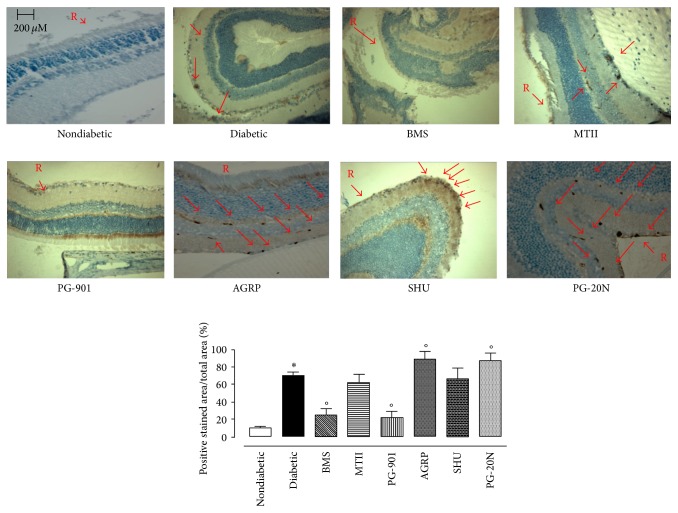
Representative immunohistochemistry after 16 weeks of diabetes for ki-67 in the retina of nondiabetic mice (nondiabetic), STZ-diabetic mice with retinopathy (diabetic) after intravitreal treatment or not with MC_1_ receptor agonist BMS-470539; MC_3_-MC_4_ melanocortin receptor agonist MTII; MC_1_ receptor antagonist agouti related protein (AGRP); MC_5_ melanocortin receptor agonist PG-901; MC_3_-MC_4_ melanocortin receptor antagonist SHU9119; MC_5_ melanocortin receptor antagonist PG20N. Percentage of positive stained area/total area with significant differences against nondiabetic mice is expressed as ^*∗*^ *p* < 0.01. Significant differences versus diabetic are expressed as °*p* < 0.01. R = retina; arrows indicate the positive immunostaining.

In line with this trend, immunohistochemistry for ki-67 showed a decrease in the percentages of positive stained area/total stained area following intravitreal injection of BMS-470539 (MC_1_ agonist, −64.2 ± 6% versus diabetic) or PG-901 (MC_5_ agonist, −68.6 ± 7%) as calculated against the values quantified in week 16 diabetic mice (Figure 7).

### 3.6. Melanocortin Receptor Activation Changes Macrophage Phenotype Polarizations

**Figure 8:**
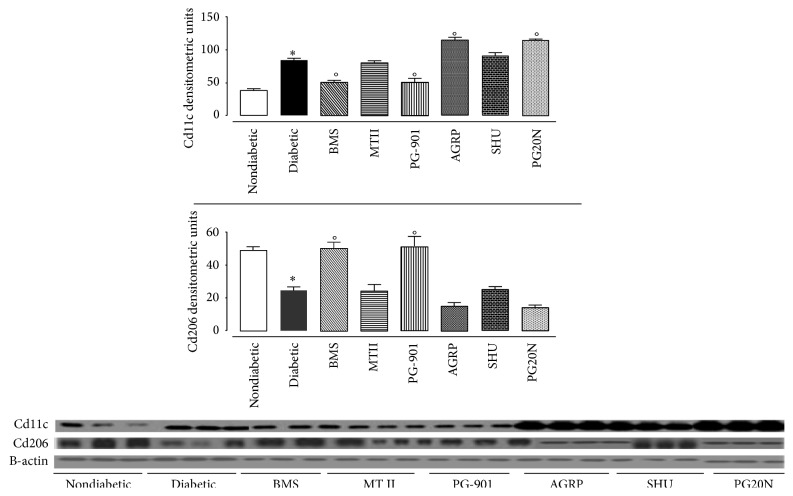
Expression of retinal Cd11c and Cd206 in eyes of mice with retinopathy. 16-week time point western blotting traces and relative densitometric units of binding to anti-Cd211 and Cd206 antibodies in the retina of nondiabetic mice (nondiabetic), STZ-diabetic mice with retinopathy (diabetic) after intravitreal treatment or not with MC_1_ receptor agonist BMS-470539; MC_3_-MC_4_ melanocortin receptor agonist MTII; MC_1_ receptor antagonist agouti related protein (AGRP); MC_5_ melanocortin receptor agonist PG-901; MC_3_-MC_4_ melanocortin receptor antagonist SHU9119; MC_5_ melanocortin receptor antagonist PG20N. Significant differences against nondiabetic mice are expressed as ^*∗*^ *p* < 0.01; significant differences versus diabetic are expressed as °*p* < 0.01.

Western blotting of retina homogenates showed that the presence of the CD11c marker for M1 macrophages was increased in diabetic mice at all time points considered, compared with healthy nondiabetic mice (Figure 8). There was a good correlation between the number of M1 macrophages and the levels of occludin in the retina with an *r* ^2^ = 0.9732. The number of M1 macrophages was further increased by intravitreal injection of the MC_1_ or MC_5_ antagonist (Figure 8). In contrast, intravitreal injection of the MC_1_ or MC_5_ agonist in diabetic mice affected by retinopathy decreased the binding for CD11c (Figure 8).

To complement these analyses, we then also assessed expression of an M2 marker, the mannose receptor CD206. Intravitreal treatment with BMS-470539 or PG-901 increased CD206 detection, when compared to diabetic mice (Figure 8), in line with the improvement of the ocular signs recorded with the FAG. This suggests an elevated presence of M2 subtype macrophages.

### 3.7. Melanocortin Receptor Activation Modulates Cytokine and Chemokine Expression Profiles

**Figure 9:**
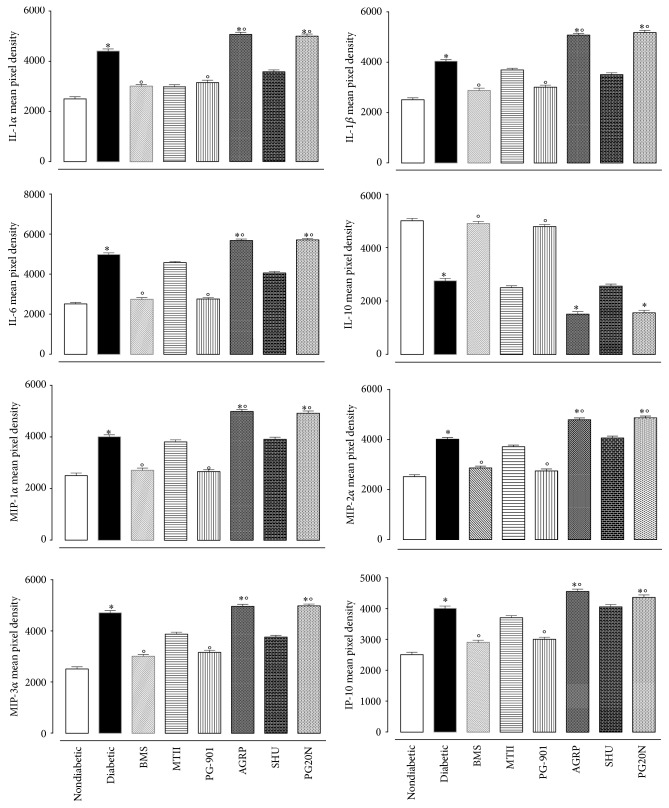
Array of cytokines and chemokines released in the retina of diabetic mice. A specific ELISA kit was used in order to quantify the levels of cytokines and chemokines after 16 weeks of diabetes in mice treated or not with the melanocortin receptor agonists/antagonists as in Figure 8. The values represent the mean ± SEM of 8–10 mice per group. Significant differences against nondiabetic mice are expressed as ^*∗*^ *p* < 0.01; significant differences versus diabetic are expressed as °*p* < 0.01.

Around 40 cytokines and chemokines were analysed using the retinal extracts. The proinflammatory cytokine IL-1*β* was increased by ~61% in response to the development of retinopathy (*p* < 0.01 versus nondiabetic mice without retinopathy; Figure 9). The melanocortin receptor antagonists AGRP and PG20N further increased expression levels for IL-1*α*, IL-1*β*, IL-6, MIP-1*α*, MIP-2*α*, and MIP-3*α* (Figure 9). For example, IL-1*β* was increased by 26 ± 2% and 28 ± 1.8%, respectively, for AGRP and PG20N (*p* < 0.01 versus diabetic). Administration of BMS-470539 or PG-901 significantly diminished IL-1*α*, IL-1*β*, IL-6, MIP-1*α*, MIP-2*α*, and MIP-3*α* in the retina (*p* < 0.01 versus diabetic; Figure 9). MTII and SHU9119 did not significantly affect the expression of these proinflammatory mediators (Figure 9).

A similar trend was observed for the proinflammatory chemokines MIP-1*α*, MIP-2*α*, and MIP-3*α*, where the highest levels were reached in diabetic mice 16 weeks after diabetes induction (Figure 9). Modulation of MC_1_ and MC_5_ receptor signaling by means of agonists and antagonists significantly modified MIP-1 *α*, MIP-2*α*, and MIP-3*α* levels (Figure 9).

Finally, and of further interest, while IL-10 levels were low in the retina of diabetic mice with retinopathy, the expression of this anti-inflammatory cytokine was significantly increased after the injection of either MC_1_ or MC_5_ agonist, for example, +64 ± 7% and +56 ± 4.2% for BMS-470539 and PG-901, respectively (*p* < 0.01 versus diabetic mice) (Figure 9).

## 4. Discussion

In this study we investigated the putative protective effect of intravitreal injection of melanocortin agonists in a model of streptozotocin- (STZ-) induced diabetic retinopathy (DR) in mice. This is in light of the incomplete and still ongoing knowledge of the pathogenetic molecular mechanism underlying DR, the lack of structural, functional, and biochemical studies in human subjects, and the need of new local therapeutic options.

The data presented here indicates that mice suffering from prolonged (16 weeks) diabetes develop retinal alterations typical of nonproliferative diabetic retinopathy (DR), such as microaneurysms with irregular vascular course and vessel leakage, appearing from 12 weeks after the onset of diabetes. These alterations were markedly reduced by the intravitreal injection of the MC_1_ and MC_5_ melanocortin receptor agonists BMS-470539 and PG-901, respectively. These compounds preserved a regular course and caliber of the vessel with no signs of leakage even 16 weeks after diabetes induction. In contrast, animals treated with the MC_1_ antagonist AGRP, or the MC_5_ antagonist PG20N, showed worsening of DR clinical signs. As early as 8 weeks after induction of diabetes, approximately 80% of mice treated with MC_1_–MC_5_ antagonists display appearance of venous loop with marked leakage, due to an increased vascular permeability, and vascular tortuosity. Therefore, these antagonists enable identification of a “protective melanocortin tone” in the eye during DR.

DR is clinically divided in two types: nonproliferative (NPDR) and proliferative (PDR) [20]. The two types exhibit typical and distinctive clinical signs. Thus, NPDR is characterized by microaneurysms, dot and blot hemorrhages, and, in severe cases, retinal microvascular damage and intraretinal microvascular abnormalities [32]. PDR, on the other hand, is characterized by abnormal retinal neovascularization due to capillary nonperfusion, and retinal ischemia [33]. Vascular changes characteristics of diabetic retinopathy in humans have been widely documented in diabetic rats, dogs, and cats [34–39], including the breakdown of the blood-retinal barrier, damage in nonvascular retinal neurons and Müller glial cells, thickening of the capillary basement membrane, reduction in the number of pericytes, and an increase in the number of acellular capillaries [34–39].

The model we used here replicates the early signs of nonproliferative DR, such as loss of retinal pericytes and capillaries, thickening of the vascular basement membrane, and increased vascular permeability [23], signs that were significantly reduced by activation of retinal MC_1_ and MC_5_ receptors. These receptors are part of the 4 receptors that are differentially expressed in the neuroretina layers [12, 16]; as MC_3_ and MC_4_ receptors are localised in the layer of retinal ganglion cells, MC_5_ receptors are expressed in the neural outer plexiform layer, whilst the retinal pigment epithelial cells express MC_1_ and MC_5_ receptors [16, 17]. They translate the actions of melanocortins in ocular immunity, development, and health, and in neurotrophism of eye tissues [18–20], together with biomolecular changes relating to cell protection. MC_3_ and MC_5_ do not appear to transduce the protective effects of melanocortin peptides, at least in these setting and with respect to the markers under analysis in our setting. DR is a pathology initiated by hyperglycemia which overall (i) increases polyol pathway flux; (ii) increases advanced glycation end-product (AGE) formation; (iii) activates protein kinase C (PKC) isoforms; and (iv) increases hexosamine pathway flux [40]. These events lead to upregulation of VEGF, insulin-like growth factor (IGF), angiopoietins (Ang-2), tumor necrosis factor-*α* (TNF-*α*), and IL-6 [41–43], responsible for a florid inflammatory response within the eye. Modulation of melanocortin receptor activity did not affect systemic glycemia, suggesting therefore local activation of protective mechanisms. Although we cannot rule out potential off target activity of the melanocortin receptor agonists that may influence the results, we propose that specific activation/deactivation of inflammatory and oxidative signaling regulated by selective melanocortin receptor agonists may constitute an alternative approach for the treatment of DR, affording protection of retinal vessels, slowing down, and/or preventing the onset of early vascular changes typical of DR.

Congruent with these effects, and the notion of localised responses, functional actions of MC_1_–MC_5_ evoked changes in the main mediators of the inflammatory response including cytokines (such as IL-1*α*, IL-1*β*, IL-6, and IL-10) and chemokines (such as MIP-1*α*, MIP-2*α*, and MIP-3*α*). These actions were married to changes of the main markers of vessel proliferation such as VEGF and ki-67. This evidence is supported by previous and almost extensive studies on melanocortin activities in a wide range of settings [14], including their protective effects in chondrocytes [44, 45] and human primary cells [46] and* in vivo* models of diseases like rheumatoid arthritis, colitis, allergic airway inflammation, or ischemia reperfusion injury [12, 16, 36, 47]. These multiple properties are likely due to the ability of melanocortins to reduce production of proinflammatory cytokines by inhibiting NF-*κ*B translocation to the nucleus as demonstrated by Manna and Aggarwal [5] or production of anti-inflammatory cytokines such as IL-10 from monocytes as demonstrated by Redondo et al. [8], Grabbe et al. [9], and ourselves here. Further support to the data presented and discussed here derives from the protective and anti-inflammatory properties of melanocortins in experimental models of exogenous uveitis [20, 48] and of retinal degeneration [19]. Retinal MC_1_–MC_5_ activation also modified the retinal macrophage population commonly associated with the released cytokines, and with the disruption of normal retina structure. In this context we observed a high level of M1 macrophages within the retina of diabetic mice that developed retinopathy, as evidenced by the western blotting performed with the specific M1 marker CD11c, with respect to the mice that developed diabetes only without retinopathy. These macrophages are notoriously characterized by a strong propensity to the production of cytokines and nitric oxide and reactive oxygen [49], which contribute to the retinal vascular damage. M2 macrophages were abundantly present into the retina after the stimulation of the MC_1_ or MC_5_ for 8 weeks. Noteworthily, these macrophages are associated with resolution of the immune-inflammatory responses into tissues activated by previous insults [50, 51] and into the consequent tissue regeneration.

Although clinically DR can be managed through the control of the metabolic glucose pathway, we conclude this study by proposing that stimulation of the endogenous melanocortin system in the eye through the local activation of MC_1_ and MC_5_ may reduce the retinal damage caused by diabetes. This may be the start of a melanocortin-based therapy for DR, especially when considering that several natural and synthetic melanocortin receptor agonists are under clinical experimentation [52, 53].

## Figures and Tables

**Table 1:** Mean glycemia levels (mg/dl) in nondiabetic and diabetic mice treated with melanocortin receptor agonists/antagonists.

Mean glycemia levels (mg/dl ± SEM)								
Weeks	Nondiabetic mice	Diabetic mice	Diabetic mice + BMS	Diabetic mice + MTII	Diabetic mice + PG-901	Diabetic mice + AGRP	Diabetic mice + SHU	Diabetic mice + PG20N
8	80 ± 8	330 ± 8	315 ± 16	345 ± 12	315 ± 17	324 ± 7	355 ± 19	335 ± 14
12	90 ± 10	335 ± 20	310 ± 15	325 ± 14	327 ± 13	354 ± 18	312 ± 10	325 ± 16
16	80 ± 12	320 ± 14	305 ± 12	355 ± 10	346 ± 24	342 ± 21	333 ± 16	340 ± 9

Intravitreal injections (5 *µ*l) every 4 weeks from the onset of diabetes with BMS = BMS-470539 (MC_1_ agonist, 33 *µ*mol); MTII (MC_3_-MC_4_ agonist, 9.3 nmol); PG-901 (MC_5_ agonist, 7.32 nM); AGRP (MC_1_ antagonist, 1 *µ*g/mouse); SHU = SHU9119 (MC_3_-MC_4_ antagonist, 9 nmol); PG20N (MC_5_ antagonist, 130 nM).
