# Corrigendum to “Activation of Melanocortin Receptors MC_1_ and MC_5_ Attenuates Retinal Damage in Experimental Diabetic Retinopathy”

**DOI:** 10.1155/2021/9861434

**Published:** 2021-03-30

**Authors:** S. Rossi, R. Maisto, C. Gesualdo, M. C. Trotta, F. Ferraraccio, M. K. Kaneva, S. J. Getting, E. Surace, F. Testa, F. Simonelli, P. Grieco, F. Merlino, M. Perretti, M. D'Amico, C. Di Filippo

**Affiliations:** ^1^Multidisciplinary Department of Medical-Surgical and Dental Specialties, Second University of Naples, 80138 Naples, Italy; ^2^Department of Experimental Medicine, Second University of Naples, 80138 Naples, Italy; ^3^Department of Clinical, Public and Preventive Medicine, Second University of Naples, 80138 Naples, Italy; ^4^The William Harvey Research Institute, Barts and The London School of Medicine, Queen Mary University of London, London EC1M 6BQ, UK; ^5^Faculty of Science and Technology, Department of Life Sciences, University of Westminster, London W1W 6UW, UK; ^6^Department of Translational Medicine, University of Naples Federico II, 80131 Naples, Italy; ^7^Pharmacy Department, University of Naples Federico II, 80131 Naples, Italy

In the article titled “Activation of Melanocortin Receptors MC1 and MC5 Attenuates Retinal Damage in Experimental Diabetic Retinopathy” [1], an error was identified in Figure 3 as raised on PubPeer [2]. Figure 3, BMS-470539 8 weeks, is the same as Figure 3, BMS-470539 16 weeks. The authors explained that this was due to a mistake during manuscript preparation and the corrected figure, as approved by the editorial board, is shown below.

## Figures and Tables

**Figure 1:**
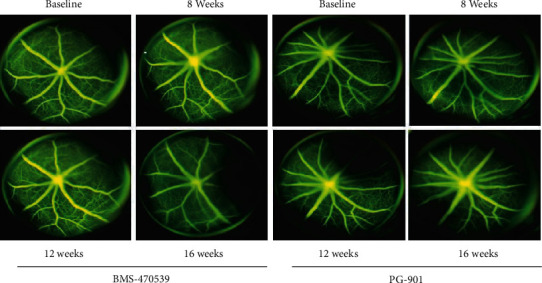
Representative pictures of FAG showed a regular course and caliber of retinal vessels without microvascular changes or vessel leakage at every time point following intravitreal injection of the MC1 melanocortin receptor agonist BMS-470539 and of the MC5 agonist PG-901. The number of mice for each group was *n* = 10 nondiabetic mice (baseline) and 8 diabetic mice with retinopathy.
